# Testing the accuracy of an observation-based classifier for rapid detection of autism risk

**DOI:** 10.1038/tp.2014.65

**Published:** 2014-08-12

**Authors:** M Duda, J A Kosmicki, D P Wall

**Affiliations:** 1 Division of Systems Medicine, Department of Pediatrics, Stanford University, Stanford, CA, USA; 2 Analytic and Translational Genetics Unit, Department of Medicine, Massachusetts General Hospital and Harvard Medical School, Boston, MA, USA; 3 Program in Medical and Population Genetics, Broad Institute of Harvard and MIT, Cambridge, MA, USA

## Abstract

Current approaches for diagnosing autism have high diagnostic validity but are time consuming and can contribute to delays in arriving at an official diagnosis. In a pilot study, we used machine learning to derive a classifier that represented a 72% reduction in length from the gold-standard Autism Diagnostic Observation Schedule-Generic (ADOS-G), while retaining >97% statistical accuracy. The pilot study focused on a relatively small sample of children with and without autism. The present study sought to further test the accuracy of the classifier (termed the observation-based classifier (OBC)) on an independent sample of 2616 children scored using ADOS from five data repositories and including both spectrum (*n*=2333) and non-spectrum (*n*=283) individuals. We tested OBC outcomes against the outcomes provided by the original and current ADOS algorithms, the best estimate clinical diagnosis, and the comparison score severity metric associated with ADOS-2. The OBC was significantly correlated with the ADOS-G (*r*=−0.814) and ADOS-2 (*r*=−0.779) and exhibited >97% sensitivity and >77% specificity in comparison to both ADOS algorithm scores. The correspondence to the best estimate clinical diagnosis was also high (accuracy=96.8%), with sensitivity of 97.1% and specificity of 83.3%. The correlation between the OBC score and the comparison score was significant (*r*=−0.628), suggesting that the OBC provides both a classification as well as a measure of severity of the phenotype. These results further demonstrate the accuracy of the OBC and suggest that reductions in the process of detecting and monitoring autism are possible.

## Introduction

Due to the significant amount of genetic heterogeneity that underlies autism spectrum disorder (ASD), autism is primarily diagnosed through behavioral evaluations. Current diagnostic instruments have been designed to measure impairments in three core developmental domains: (1) language and communication; (2) reciprocal social interactions; and (3) restricted, repetitive behaviors. The most widely used of these instruments is the Autism Diagnostic Observation Schedule-Generic (ADOS-G)^1^ and the updated version ADOS-2.^2^ Clinically trained professionals administer the ADOS exam to elicit specific types of responses during a series of structured activities. The exam is divided into four modules based on language and developmental level of the subject, ensuring coverage for a wide variety of ages and behavioral manifestations. Module 1, which contains 10 activities and 29 items, is the most commonly used for assessment of younger children because it is tailored to individuals with little or no language. Total time for administration and scoring of the ADOS exam can range from 60 to 90 min.

Recently, Western Psychological Services published an updated exam and scoring algorithm, termed the ADOS-2 (http://www.wpspublish.com/).^2^ This revised instrument examines behaviors in two distinct domains, social affect and restricted, repetitive behaviors, and contains five modules suited for various developmental groups ranging from nonverbal toddlers to high-functioning adults. ADOS-2 represents an increase in overall accuracy from ADOS-G, notably increasing specificity in identifying spectrum cases from non-spectrum controls in lower functioning populations as well as increasing comparability between modules.^2, 3, 4^ The ADOS-2 exam utilizes the same 10 activities as the ADOS-G, however it adds four new items and implements revisions to 11 of the preexisting items. This new scoring algorithm also provides instructions for calculating the ADOS-2 Comparison Score, a metric for gauging autism severity. To calculate this severity score, subjects are divided into age- and language-specific cells, where the overall total corresponds to a comparison score on a scale of 1–10 (10 representing the highest severity of autism-related symptoms).^4, 5, 6^ Total administration and scoring time of the ADOS-2 is estimated to be roughly equal to or slightly longer than the ADOS-G, due to additional scoring components.

The length of the ADOS exam and the need for administration in a clinical facility by a trained professional both contribute to delays in diagnosis and unequal coverage of the population needing attention.^7^ The diagnostic facilities and trained clinicians tend to be geographically clustered in major metropolitan areas and far outnumbered by the individuals in need of evaluation. Diagnostic assessments also often require referrals from a child's primary care physician, and due to time and resource constraints during pediatric well visits, initial autism screenings are not consistently conducted.^8^ Families may face waiting periods as long as 13 months between initial screening and clinical diagnosis,^9^ or even longer if part of a minority population or lower socioeconomic status.^10^ Largely due to these waiting times, the average age of diagnosis in the United States remains above 4 years and an estimated 27% remain undiagnosed at 8 years of age.^7^ The delays in diagnosis directly translate into delays in the delivery of speech and behavioral therapies that have significant positive impacts on a child's development, especially when delivered early in life.^11^ By 5 years of age, the benefits of therapy are generally considered to be lower than if delivered earlier in life. Streamlining the process to enable a diagnosis within weeks or even days of initial suspicion rather than months could lead to significant improvements in outcomes.

Considering the enormous benefit of delivering interventions and therapies early in development as well as the growing prevalence of autism in the United States^12,13^ and across the globe, there is a significant and immediate need for abbreviated methods to detect ASD rapidly and with high accuracy. Several attempts have been made to develop fast methods for behavioral identification of autism, including the Social Responsiveness Scale,^14^ Social Communication Questionnaire^15^ and the Modified Checklist for Autism in Toddlers.^16^ Although these methods have various uses and considerable value for abbreviated screening, longer and more detailed diagnostic instruments, including the ADOS and the Autism Diagnostic Interview-Revised (ADI-R),^17^ remain the most used tools for assistance with the clinical diagnosis of autism, due to their comparatively high degrees of diagnostic accuracy.^18,19^

By utilizing a machine-learning approach, we created a machine-learning classifier that shows promise for distinguishing cases of autism from non-spectrum and that exhibited high accuracy—99.7% sensitivity and 94% specificity—in initial tests.^20^ The classifier currently includes eight behaviors identified through machine learning on ADOS score sheets, 72% fewer behaviors than on ADOS-G and 76% fewer than on ADOS-2. Since the initial study, the sizes of the research data sets have grown, enabling better opportunities to test its sensitivity and specificity. Here we test the accuracy of this classifier, the observation-based classifier (OBC), in a cohort of archival score sheets of over 2600 subjects, including more than 280 assessments of non-spectrum controls. We measure sensitivity and specificity against the original gold-standard Autism Diagnostic Observation Schedule-Generic (ADOS-G) scoring algorithm, the revised ADOS-2 algorithm, and the best estimate clinical diagnoses. We also assess the correspondence of our classifier with the comparison score severity scale outlined by ADOS-2.

## Materials and methods

### Data samples

We acquired 2616 complete ADOS Module 1 score sheets from the Boston Autism Consortium, Simons Simplex Collection version 14, Simons Variation In Individuals Project, Autism Genetic Resource Exchange, and National Database for Autism Research data sets (Table 1). Domain scores were computed according to the ADOS-G diagnostic algorithm, and individuals were classified into categories of autism, autism spectrum and non-spectrum based on meeting specified thresholds in each domain. For our purposes, the categories of autism and autism spectrum were combined to test the ability of the classifier to distinguish spectrum cases from non-spectrum controls.

### Algorithms

#### Observation-based classifier

We developed the observation-based classifier (OBC) as a short quantitative tool for autism classification, which exhibited high accuracy in our initial tests.^20^ The classifier presently contains eight behaviors (Table 2) that are often impacted in children with autism, including eye contact, imaginative play and reciprocal communication. The OBC utilizes an alternating decision tree algorithm (ADTree)^20, 21^ to compute a quantitative score that indicates the class (spectrum or non spectrum) as well as the confidence in the classification. Following from the statistical properties of the ADTree, values closer to zero are considered lower confidence scores.

#### ADOS-G

ADOS scoring instruments were purchased from Western Psychological Services (http://www.wpspublish.com/). The standard ADOS-G Module 1 algorithm combines questions from the social and communication domains of the 29-item exam. A classification of autism requires a score of 7 or more in the social domain, a score of 4 or more in the communication domain and a combined social-communication total of at least 12. A classification of autism spectrum requires a score of 4 or more in the social domain, a score of 2 or more in the communication domain and a minimum social-communication total of 7. Otherwise, a classification of non-spectrum is provided.

#### ADOS-2

We used the modified ADOS Module 1 algorithm for computing ADOS-2 scores. Since the ADOS-2 and ADOS-G both utilize the same 16 algorithm items, it was possible to apply the ADOS-2 algorithm on ADOS-G score sheets. Of these 16 common items, 9 items were revised to provide additional clarification to the administrator and only 3 items (B5, D1 and D2) exhibited coding changes in the ADOS-2 algorithm. In all three cases, behaviors that were originally captured in the code of 2 were branched into a new code of 3. For both exams, responses coded as 3s are re-coded to 2s in the scoring algorithm, and thus these changes did not affect the overall calculation of ADOS-2 algorithm scores. The scoring algorithm used is dependent upon the verbal level of the child (either some words or few to no words), which is based on the answer to question A1. The algorithms combine items from the social affect and restricted and repetitive behaviors domains into a total score on which the cutoffs are based. In the ‘some words' algorithm, a minimum score of 8 indicates autism spectrum and a score of 12 or more results in a classification of autism. In the ‘few to no words' algorithm, a minimum of 11 is required for an autism spectrum classification and a total of 16 or more indicates classic autism. In both algorithms, individuals that do not meet the lower thresholds are classified as non-spectrum.

#### Comparison score

The ADOS-2 diagnostic algorithm also provides an algorithm for computing the comparison score, a measure of the severity of autism-related symptoms. To derive the comparison score, individuals are separated into cells based on age and verbal level. Within each age-language cell, ADOS-2 total scores are matched to their corresponding comparison scores. The comparison score ranges from 1–10, where 1 indicates minimal-to-no evidence of autism-related symptoms and 10 indicates a high level of impairment.

### Comparisons

Because the data were ordinal and not normally distributed, we used Spearman rank correlation to compare scores provided by the OBC algorithm with the ADOS results. Sensitivity and specificity were computed to assess the correspondence of classification outcomes between the OBC and ADOS as well as the best estimate clinical diagnosis.

## Results

We analyzed a total of 2616 ADOS score sheets, 2333 of which fit the criteria for a spectrum diagnosis (autism=1884, ASD=449), and 283 of which fit the criteria for a non-spectrum diagnosis as defined by the ADOS-G diagnostic algorithm. It is important to note that a majority of the non-spectrum individuals were recruited to the study with suspicion of risk for an autism spectrum disorder or other learning delay, and thus likely constitute symptomatically challenging controls. For instances where IQ was available, the average IQ was consistent with intellectual disability in both the autism (59.5±19.1, *N*=144) and non-spectrum (69.4±17.6, *N*=25) individuals. The cohort studied was 76.8% male, 18.5% female and 4.7% unknown gender, and the average age at testing was 6.21 years (±4.25 years).

Table 3 shows the overall sensitivity and specificity of the OBC in comparison to both the ADOS-G and ADOS-2 algorithms. OBC had high overall correspondence with the ADOS-G (*r*=−0.814) and ADOS-2 (*r*=−0.779) diagnostic instruments. Sensitivity of the OBC was >97% against both the ADOS-G and ADOS-2, and the specificity was also high against both gold-standard instruments (77% and 84%, respectively).

### False positives

**Figure 1:**
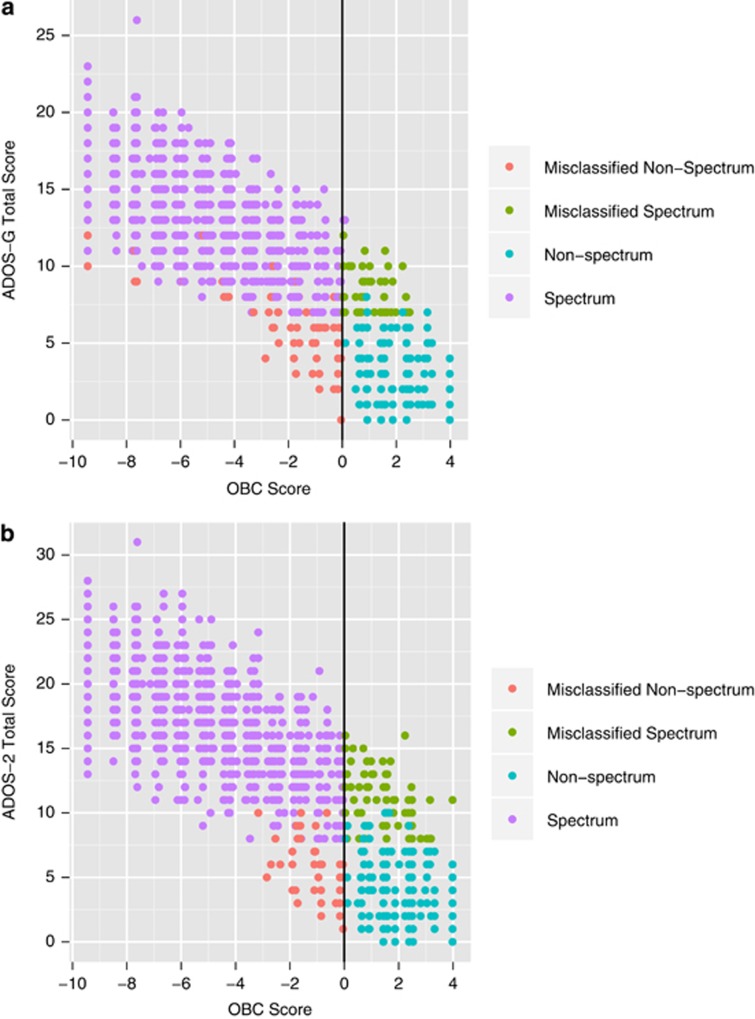
Correspondence between the observation-based classifier (OBC) score and the Autism Diagnostic Observation Schedule (ADOS). Correlation to the original ADOS-G (**a**) and the revised ADOS-2 (**b**) algorithm was high, *r*=−0.814 and −0.779, respectively.

Fifty-three individuals were misclassified as non-spectrum by the OBC when the ADOS-G classified them as spectrum cases (Figure 1a). Of these, only two met thresholds for autism classifications. Of the remaining 51, 36 had borderline domain and total scores for autism spectrum classification and 1 was labeled within the research database as having a clinical diagnosis of non-spectrum.

Sixty-nine of 2317 spectrum cases were misclassified by the OBC in comparison to ADOS-2 (Figure 1b). Twenty-four of these misclassified individuals met cutoffs for an ADOS-2 classification of autism, and 22 of the remaining 45 had borderline ADOS-2 totals for autism spectrum classification. One individual had a documented clinical diagnosis of non-spectrum.

### False negatives

Sixty-four individuals were misclassified as spectrum by the OBC (Figure 1a) when compared with the results from ADOS. Of those, eight had a diagnosis of an ASD provided by a clinician. Thirty-five of these misclassified individuals met two out of three diagnostic criteria for an ADOS-G spectrum classification.

A smaller number (*n*=40) of controls were misclassified as spectrum by the OBC when compared with the results from ADOS-2 (Figure 1b). One had a documented autism diagnosis and six had OBC scores that were closer to zero and therefore lower in confidence.

### Comparison to clinical diagnosis

Due to differences in data capture and record annotation, we were able to obtain best estimate clinical diagnoses for only a subset of individuals (for ASD *n*=789 and non-spectrum *n*=18). Seventeen of the controls had non-spectrum clinical diagnoses including language delay (*n*=14), developmental coordination disorder (*n*=11), mental retardation (*n*=4) and ADHD (*n*=2).

OBC showed 97.1% sensitivity, 83.3% specificity and 96.8% total accuracy when compared with the best estimate clinical diagnosis (Table 3). These values were comparable to the sensitivity and specificity computed for the ADOS algorithms on the same population, and comparable to the performance of the OBC when evaluated against the ADOS-G and ADOS-2 in the complete set of 2616 individuals.

### Comparison score

**Figure 2:**
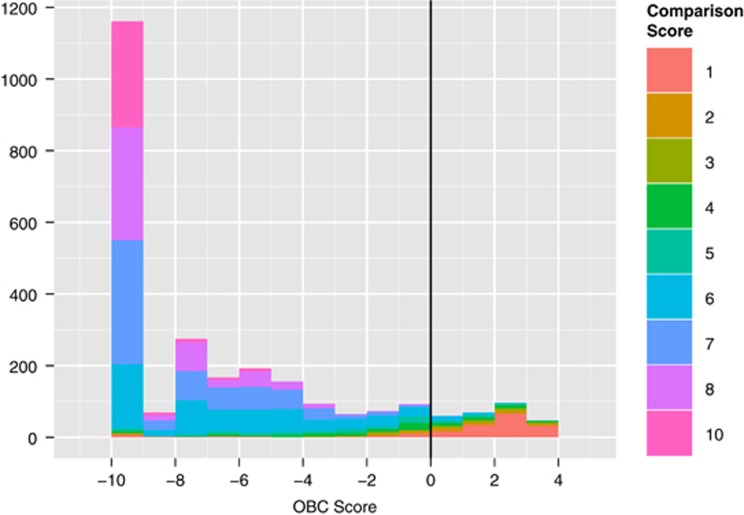
Distribution of the observation-based classifier (OBC) scores in our sample (*n*=2616), colored by the ADOS-2 comparison score (CS), a proxy for measuring autism symptom severity. A majority (86.3%) of our sample was classified in the moderate (5⩽CS⩽7) to severe (8⩽CS⩽10) range, and the OBC and CS scores were found to be significantly correlated (*r*=−0.628).

Figure 2 shows the distribution of autism phenotype severity scores versus OBC scores; a large percentage of our sample consisted of children with classic autism, possibly due to the inclusion criteria of the research collections used in our study. The correspondence between OBC score and the comparison score is significant (*r*=−0.628), indicating that the OBC score not only reflects confidence in the classification, but may also reflect the severity of the autism phenotype.

## Discussion

The purpose of this study was to independently validate the OBC—a machine learning classifier aimed at rapid detection and quantification of autism using a small number of behaviors—on a large collection of data from children with and without autism. We compiled 2616 ADOS Module 1 score sheets from five different sources to assess the OBC against the ADOS-G and ADOS-2 diagnostic algorithms. The classifier performed with >95% statistical accuracy and demonstrated high positive and negative predictive values when compared with ADOS-G, ADOS-2 and the best estimate clinical diagnosis (Table 3). These results support the hypothesis that the OBC has both high recall and precision in a reasonably diverse sample of cases with and without autism, including children with other learning and developmental delays.

The accuracy of the OBC was slightly lower than in our initial analysis (95% as compared with 97%), with a drop in specificity from 94% in the initial study to ~84% in the present work. The drop in specificity was likely due to the small number of controls used in the original training and testing data.^20^ However, the present calculations show an overall improvement in accuracy, including a large increase in specificity in comparison with existing screening tools such as the Social Communication Questionnaire (80% sensitive, 56% specific),^15^ the Social Responsiveness Scale parent (91% sensitive, 8% specific) and the Social Responsiveness Scale teacher (84% sensitive, 41% specific) reports.^14^ Less than 5% of all tested cases were misclassified by the OBC and 78% of the misclassified individuals were given a low OBC score <|2|, one that would be flagged as low confidence and warranting further assessment, such as in scenarios where the OBC is used for initial screening or clinical triage of children with risk of developmental delay.

Our OBC results also showed significant correlation to the ADOS-2 comparison score (*r*=−0.628), supporting the hypothesis that the OBC reflects severity of the autism phenotype. This suggests the possibility that the OBC can provide information for rapid classification as well as data useful for triage of at-risk individuals on long waiting lists at clinical centers. For example, use of the OBC as a web-based assessment in advance of a clinical visit may enable clinicians to quickly prioritize patients according to symptom severity, scheduling shorter, more immediate diagnostic appointments for individuals that can be clearly identified as on or off the autism spectrum, and allowing longer time periods for deeper evaluation of children that exhibit clinically challenging symptoms.

Although more testing is needed to determine if (and how) the approaches described here can have clinical value, there is little doubt that the field needs novel methods for initial screening, diagnosis and monitoring that can reach a larger percentage of the rising autism population. Here we demonstrate the accuracy of a short classification algorithm that focuses on eight behaviors. In a recent related study, we demonstrated that autism characteristics can be captured and correctly identified in unstructured home videos in under five  minutes by trained, nonclinical raters.^20^ Taken together these studies suggest that web-accessible platforms for rapid measurement of autism risk and tracking of children with developmental concerns could be within reach. Such mobilized approaches would serve to improve the efficiency with which diagnostically relevant information is delivered to clinicians, helping to shorten screening and diagnostic processes overall and potentially enabling more families to receive care far earlier and during timeframes when interventions have the most positive benefits. More work is necessary to demonstrate feasibility, including further clinical validation, testing of mobile use by parents in adavance of clinical visits, and the utility of the tool as a method for clinical triage.

## Figures and Tables

**Table 1:** Data set breakdown by source

	*AC*		*SSC*		*VIP*		*AGRE*		*NDAR*	
	*Spectrum*	*Non-spectrum*	*Spectrum*	*Non-spectrum*	*Spectrum*	*Non-spectrum*	*Spectrum*	*Non-spectrum*	*Spectrum*	*Non-spectrum*
Sample size	140	5	505	11	10	17	803	14	875	236
Average age (years)	4.9	2.5	6.1	8.6	5.8	6.1	5.3	6.9	6.8	8.1

Abbreviations: AC, Autism Consortium; AGRE, Autism Genetic Resource Exchange; NDAR, National Database for Autism Research; SSC, Simons Simplex Collection; VIP, Variation In Individuals Project. Score sheets from the Autism Diagnostic Observation Schedule-Generic (ADOS-G) Module 1 were acquired from the Boston AC, SSC, Simons VIP, AGRE and NDAR data sets. Listed are the total numbers of individuals classified as spectrum (autism and autism spectrum) and individuals classified as non-spectrum by ADOS represented in each of the five data sets.

**Table 2:** The eight behaviors presently evaluated by the observation-based classifier together^20^

*Behavior targeted*	*Core domain*
Frequency of vocalization directed to others	Communication
Eye contact	Social interaction
Social smile	Social interaction
Shared enjoyment in interaction	Social interaction
Showing	Social interaction
Initiation of joint attention	Social interaction
Functional play with objects	Play
Imagination/creativity	Play

**Table 3:** Overall sensitivity and specificity of the observation-based classifier computed against the ADOS-G and ADOS-2 algorithm scores and best estimate clinical diagnosis

	*ADOS-G*		*ADOS-2*		*Clinical diagnosis*	
	*Spectrum*	*Non-spectrum*	*Spectrum*	*Non-spectrum*	*Spectrum*	*Non-spectrum*
*OBC*						
Spectrum	2280	64	2304	40	766	3
Non-spectrum	53	219	69	203	23	15
			
Sensitivity	0.977		0.971		0.971	
Specificity	0.774		0.835		0.833	
Accuracy	0.955		0.958		0.968	
PPV	0.973		0.983		0.996	
NPV	0.805		0.746		0.395^a^	

Abbreviations: ADOS-G, Autism Diagnostic Observation Schedule-Generic; NPV, negative predictive value; OBC, observation-based classifier; PPV, positive predictive value. Sensitivity was comparable across the three baselines for comparison; specificity was highest (>83%) when compared with ADOS-2 and best estimate clinical diagnosis.

a Based on limited number of non-spectrum individuals.
